# Attenuation of Some Inflammatory Markers by Endurance Training in the Spinal Cord of Rats with Diabetic Neuropathic Pain

**DOI:** 10.1155/2022/6551358

**Published:** 2022-05-18

**Authors:** Abdolhamid Habibi, Asma Taheri, Saba Habibi

**Affiliations:** ^1^Department of Exercise Physiology, Faculty of Sport Sciences, Shahid Chamran University of Ahvaz, Ahvaz, Iran; ^2^Graduate of Exercise Physiology, Shahid Chamran University of Ahvaz, Ahvaz, Iran; ^3^School of Medicine, University of Turin, Turin, Italy

## Abstract

Nervous inflammation is an important component of the pathogenesis of neurodegenerative diseases including chronic diabetic neuropathic pain. In order to obtain a decrease in the progression of diabetic neuronal damage, it may be necessary to examine therapeutic options that involve antioxidants and anti-inflammatory agents. The aim of this study was to investigate the attenuation of inflammatory factors with endurance training in the spinal cord of rats with neuropathic pain. Thirty-two 8-week-old male Wistar rats (with a weight range of 204 ± 11.3 g) were randomly divided into 4 groups (*n* = 8), including (1) diabetic neuropathy (50 mg/kg streptozotocin intraperitoneal injection), (2) diabetic neuropathy training (30 minutes of endurance training at 15 meters per minute, 5 days a week for 6 weeks), (3) healthy training, and (4) healthy control. After confirmation of diabetic neuropathy by behavioral tests, training protocol and supplementation were performed. The NLRP3, P38 MAPK, TNF-*α*, and IL-1*β* gene expressions were measured by a real-time technique in the spinal cord tissue. One-way analysis of variance and Tukey's post hoc test were used for statistical analysis. Endurance training reduced the sensitivity of the nervous system to thermal hyperalgesia and mechanical allodynia; also, compared to the diabetic neuropathy group, the gene expressions of NLTP3, P38 MAPK, TNF-*α*, and IL-1*β* were significantly reduced by endurance training (*P* < 0.05). Endurance training modulates NLRP3, P38 MAPK, and TNF-*α*, IL-1*β* gene expressions and improves the sensitivity of nociceptors to pain factors. Accordingly, it is recommended to use endurance training to reduce neuropathic pain for diabetics.

## 1. Introduction

Diabetic neuropathy is one of the most well-known neurological diseases due to diabetes and its prevalence is 50 to 60%. Diabetic neuropathy is associated with structural changes in peripheral nerves, so due to this axonal atrophy, demyelination, reduced neurological cords, decreased nerve blood flow, and declined repair or reproduction process in neurological cords occur; these complications can cause significant consequences, including pain and lack of analgesia in the patient [1, 2]. Neuropathic pain is one of the most common symptoms of diabetic neuropathy, from which about 30% of patients suffer; while the rest of patients may experience a negative sense such as insensibility and numbness. Neuropathic pain is characterized by features such as thermal hyperalgesia and mechanical allodynia. While there are several theories of the etiological cause of diabetic neuropathic pain, peripheral and neuronal glial cell damage has been discovered to cause diabetic neuropathic pain through inflammation [2]. Recent studies have examined the inflammatory and pre-apoptotic role of NLRP3 inflammasomes in diabetes and its complications [3–5].

NLRP3 inflammasome is a multiprotein complex that coordinates the innate immune responses by activating caspase-1 and the induction of pre-inflammatory cytokines [6]. Pre-inflammatory cytokines such as IL-1*β* and TNF-*α* induce neuropathic pain [7]. Inflammation is a physiological response that results in widespread chemical and cellular reactions in the body. In various diseases, nerve inflammation is a stimulator of immune inflammatory reactions of agents such as IL-6, TNF-*α*, and IL-10, leading to an increase in active astrocytes and the number of microglia and activation of the inflammatory enzyme system, such as iNOS and COX, and affects the structure and function of the neurons [8]. The results of recent studies indicate that little enquiry has been conducted on the mechanism of endurance training as a nondrug and noninvasive therapeutic intervention on diabetic neuropathic pain at lower cellular levels. In order to obtain a decrease in the progression of diabetic neuronal damage, it may be necessary to examine therapeutic options that involve antioxidants and anti-inflammatory agents. Despite all of the pharmacological therapies that have been proposed (e.g., nonsteroidal analgesic drugs and opioids), most have been found to be not fully effective in treating or preventing the progression of diabetic neuropathic pain and may still have some negative side effects.

Therefore, the purpose of this study was to investigate whether endurance training has any effect on reducing inflammation markers in the spinal cord of rats suffering from neuropathic pain. The male Wistar rats were randomly assigned into four different groups: (1) diabetic neuropathy, (2) diabetic neuropathy training, (3) healthy training, and (4) healthy control. Furthermore, the neuropathic pain behavioral tests were carried out every week until the end of the endurance training protocol in order to assess the long-term effects of training. Afterward, the results of the experiment are discussed in the sections that follow.

This paper includes following sections. A clear description of the topic is provided in the “Introduction,” focusing on the most important theme of the paper. For readers to make sense of the discussion, the necessary background information is given in the “Literature Review” section. We present information from a variety of sources relevant to the significance of this paper in the following section. The section “Materials and Methods” describes the design of the experiment in detail. It is the purpose of this section to justify the research by either demonstrating clearly how the research was conducted or by providing a breakdown of the homework. Detailed results are gathered and summarized in a section entitled “Results”. The report is unbiased and provides accurate information. A brief summary of the major claims from previous sections is provided along with comparisons to other studies. The section titled “Conclusion” is the last step, which reinforces or interprets the major points in a way that goes far beyond simple summarization. Taking into account the complications raised by the study, further research is recommended.

## 2. Literature Review

Dorsal root ganglion (DRG) neurons are the location of primary sensory neurons, vulnerable to toxins and systemic metabolic disorders [9]. Previous studies show that the activation of inflammation of NLRP3 and DRG at the downstream results in neuropathy, spinal disk herniation, and mechanical allodynia in type 2 diabetic rodents [10–12]. Also, P38 kinases (P38 MAPK) are associated with several neurological disorders, especially when they are associated with neural inflammatory responses. This is largely based on their prominent role in the production and release of cytokines from immune cells, including microglia [13]. At present, sustainable blood glucose control and pain management are the only adjustable treatments for diabetic neuropathic pain, which requires new and effective treatments [1]. In [14], the authors have presented a new model for stochastic modeling of GREB1 gene transcription. To investigate the interactions of both parameters with TNF-∗. In [15], calculations of binding free energy, molecular docking, and molecular dynamics simulations have been combined. On the other hand, exercise as a nonpharmacological strategy has been considered as a noninflammatory factor to reduce the complications of diabetes [16]. In their research, McFarlin et al. showed that long-term activity and active lifestyle can have anti-inflammatory effects [17]. In another study, it was shown that exercise training can decrease neuropathic pain and decline inflammatory markers IL-1 *β* and TNF-*α* in a spinal cord injury [18]. Endurance training with increasing levels of thermal shock proteins, reducing levels of active oxygen species (ROS), releasing endogenous opioids, and increasing the expression of neurotrophins can reduce the levels of oxidative stress markers and prevent the progressive degradation of sensory neurons and so reduce the sensitivity of nociceptors to pain factors [19].

## 3. Materials and Methods

### 3.1. Experimental Animals and Grouping

In the present experimental study, thirty-two 8-week-old male Wistar rats with a weight range of 204 ± 11 grams were provided from the laboratory animal proliferation center of Jundishapur University of Medical Sciences in Ahvaz and were divided into four groups. The animals were maintained in polycarbonate cages in a temperature of 22 ± 2°C under 12 : 12 hours of light-dark cycle and had ad libitum access to water and special food. After a week of adaptation with the laboratory environment, familiarization with the treadmill, and manipulation, rats were randomly divided into 4 groups (*n* = 8), including (1) diabetic neuropathy (50 mg/kg streptozotocin intraperitoneal injection), (2) diabetic neuropathy training (30 minutes of endurance training at 15 meters per minute, 5 days a week for 6 weeks), (3) healthy training, and (4) healthy control. In the present study, all ethical principles of working with animals were approved by Shahid Chamran University of Ahvaz with the code number: EE/1400.2.24.32891/scu.ac.ir.

### 3.2. Induction of Diabetes

After completing the familiarization protocol and following 12 hours of fasting, diabetes was induced with intraperitoneal injection of STZ (Sigma, St. Louis, MO) dissolved in 0.05 M citrate buffer with a pH of 4.5 at a dose of 50 mg kg/body weight to create type 1 diabetes [20, 21]. Nondiabetic rats received the same volume of 0.05 M citrate buffer with a pH of 4.5 peritoneally. After 48 hours of injection, a small injury was created on the tail vein by a lancet, then a drop of blood was placed on a glucometer tape, and the tape was measured by a glucometer (Glucotrend 2, Roche Company). Rats with blood glucose over 250 mg/dl were considered as diabetic. To ensure that blood glucose did not return during the training protocol, blood sugar was measured every week as well as at the end of the period [22].

### 3.3. Behavioral Tests

Before inducing diabetes, animals were subjected to test (twice for each test) to adapt for behavioral tests. Two weeks after induction of diabetes, neuropathic pain tests were performed as indicators of pathological conditions of diabetic neuropathy and to confirm the rate of neuropathic pain in all groups [23, 24]. In order to investigate the long-term effects of training, the neuropathic pain behavioral tests were carried out each week until the end of the endurance training protocol. The behavioral tests were administered between 7 : 00 and 10 : 00 am to avoid interventional factors, such as stress-induced antinociceptive effects [24].

### 3.4. Hot Plate Test

To measure the thermal pain threshold (thermal hyperalgesia), a hot plate test was used based on the Walf and McDonald method [25]. To perform this test, the MH-9500 model hot plate (made by Borj Sanat Azma Co.) was used, which has a metal plate with a diameter of 19 cm and a Plexiglass case (30 × 25 × 25 cm). The device was equipped with a timer and thermostat. The severity of the temperature of the device was adjusted at 52 ± 2°C. Before performing the test, rats were placed on the device for 2 minutes; then the device was turned on to set the temperature of the device to the desired temperature. The animal was placed on the hot plate and simultaneously, the timer was turned on.

The time when the animal started to lick, raise, or shiver the leg was considered as the endpoint and the index of pain, and then the timer was immediately stopped and the animal was allowed to get out of the device. The paw withdrawal latency or the time interval of the start of the animal on the hot plate until the emergence of response to the pain was measured in seconds in three steps of 5 minute intervals in different groups, and their average was recorded as the latency time. The cut-off time of animal response to the hot plate was considered to be 30 seconds.

### 3.5. Mechanical Allodynia Test

In order to measure mechanical allodynia, animals were placed on a wired network and inside a Plexiglass chamber of 20 × 20 and 30 cm height. In order to get used to the new environment, the animals were introduced into a transparent chamber and placed on a latticed plate 30 minutes before the test. In order to measure mechanical allodynia, various Von Frey filaments, manufactured by Stoelting Inc., were used in the range of 2 to 60 grams (60, 26, 15, 10, 8, 6, 4, 2) to measure the skin sensitivity to contact stimulation. Each test started with the lowest weight filament, and if there was no response, higher weight filaments were gradually used. Also, if two consecutive responses (i.e., animal's lifting leg) were observed, the same weight was recorded as the paw withdrawal threshold (PWT) and the test ended. In contrast, if the animal did not respond to any filament, including filament 60, number 60 would be considered as the response threshold. Each experiment was repeated three times with at least three-minute intervals, and their average was considered as the paw withdrawal threshold (PWT) [26].

### 3.6. Endurance Training Protocol

After ensuring diabetic neuropathy in male rats, the endurance training protocol was implemented for six weeks. In the present study, the endurance training protocol was carried out based on the study of Chang Huan et al. First, in order to get used to the laboratory conditions, the treadmill, and manipulation, animals ran on the treadmill at a speed of 10 meters per minute for 10–15 minutes 5 days a week. Then, the training groups of diabetic neuropathy training and healthy training were subjected to the treadmill training, 5 sessions per week for 6 weeks [27]. The speed and duration of the treadmill training was increased gradually each week, and rose from 10 meters per minute for 10 minutes in the first week to 10 meters per minute for 20 minutes in the second week, 14–15 meters per minute for 20 minutes in the third week, 14–15 meters per minute for 30 minutes in the fourth week, and eventually 17–18 meters per minute for 30 minutes in the fifth and sixth weeks. In order to keep the achieved adaptation level, all training variables were kept constant in the final week (i.e., week 6). All training sessions were held at the end of the animal's sleep cycle between 16 : 00 and 18 : 00 hours.

### 3.7. Sample Extraction and Measurement Method

At the end of six weeks of the training program, rats were anesthetized by intraperitoneal injection of a combination of ketamine (90 mg/kg) (mg/kg10) and xylazine 48 hours after the last training session. To study biochemical variables, under sterile conditions and according to the Golderd and Chopin (1977) procedure [28], a spinal segment containing the posterior part of the spinal cord from the L4 to L6 level, which is related to spinal segments in male Wistar rats in the T13-L1 vertebrae in the spinal cord, was immediately identified. It was then separated from the spinal cord by incision in the lowest possible part. Then, using the central channel as an indicator, the spinal cord was separated into the anterior and posterior parts. The posterior region was kept frozen as the sample in −80°C; also, the samples were kept frozen in −80°C until molecular tests were performed.

### 3.8. Real-Time PCR

Using a QIAzol Lysis Reagent kit, about 50 mg of the spinal cord tissue was homogenized to extract total RNA in a ratio of 1 to 10. In order to take the protein components, the product was centrifuged at 4°C at 12000 g for 10 minutes. Then, it was mixed up with chloroform solution in 1 to 0.5 ratio and shaken for 15 seconds. The product was centrifuged at 4°C at 12000 g for 15 minutes, and the mineral and aquatic components were separated. The RNA content was taken and mixed with isopropanol solution at 1 to 0.5 ratio and was kept at room temperature for ten minutes and was then centrifuged at 4°C at 12000 g for 10 minutes The pellet containing RNA was washed in ethanol solution and dissolved in 20 *μ*L RNase-free water. The concentration of RNA was measured. Given a ratio of 260 to 280 in accordance with the Eppendorf Germany Company, a range of 1.8–2 was considered as a desirable concentration.

The synthesis of a single cDNA was performed using the primer (Oligo DT MWG-Biotech, Germany) and reverse transcription enzyme (Fermentas), based on the relevant protocol. The RT-qPCR technique was used to confirm the GDNF gene expression quantitatively. Each PCR reaction was performed using a PCR Master Mix Applied Biosystems apparatus; also, SYBR Green was applied on ABI Step One (Applied Biosystems, Sequence Detection Systems, Foster City, CA) according to the manufacturer's protocol. 40 cycles were considered for each real-time PCR cycle, and the temperatures of each cycle consisted of 94°C for 20 seconds, 60–58°C for 30 seconds, and 72°C for 30 seconds. Meanwhile, GAPDH was used as the control gene. The expression ratios of the studied genes in this research were evaluated by a threshold cycle (CT^3^) comparison method. Using data in the formula *R*=2^−(ΔΔ*CT*)^, the expression of the target gene was normalized with the reference gene and the expression of genes in the healthy group was considered as a calibrator (see Table 1).

### 3.9. Statistical Analysis

The Kolmogorov–Smirnov test was used to determine the normality of the data. To determine the significance of the difference between the groups, one-way analysis of variance was used; in the case of significant differences, Tukey's post hoc test was used to determine the intergroup differences between the mean of the groups. Data were analyzed using SPSS (version 22) at a significance level of 0.5 (*P* < 0.05).

## 4. Results

### 4.1. Data Collection

The results showed that the initial weight of the groups did not differ significantly with each other, but in the final weeks of the study, the mean weight changes of rats in the diabetic neuropathy groups were significantly lower than those of the healthy control group (*P* < 0.05). Also, the mean weight changes in the diabetic neuropathy training and healthy training groups showed a significant increase compared to the diabetic neuropathy group in the sixth week (*P* < 0.05) (see Table 2).

After induction of diabetes, blood glucose levels increased significantly in the diabetic neuropathy groups (*P* < 0.05), and this difference was significant until the end of the research period compared to the healthy control group (*P* < 0.05); also, blood glucose levels in the diabetic training group in the end of the training protocol were significantly lower than those of the diabetic neuropathy group (*P* < 0.05) (Table 2).

### 4.2. Results of the Presented Experiment

**Figure 1:**
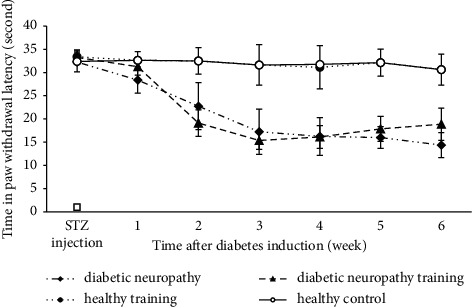
Changes in paw withdrawal latency in the thermal hyperalgesia test of different groups.

The average delay time in paw withdrawal latency in the hot plate test two weeks after the induction of diabetes was significantly lower in the diabetes neuropathy groups compared to the healthy groups (*P* < 0.05). Also, in the final weeks of implementation of the endurance training protocol, the average delay time in paw withdrawal latency in the thermal hyperalgesia test was significantly higher in the diabetic neuropathy training group compared to the diabetic neuropathy group (*P* < 0.05) (Figure 1).

**Figure 2:**
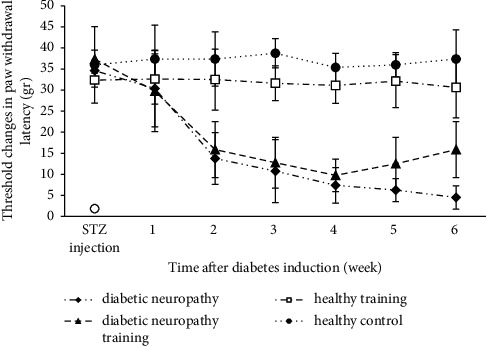
Changes in paw withdrawal latency in the mechanical allodynia test of different groups.

Two weeks after the induction of diabetes, the average threshold changes in the paw withdrawal latency in the mechanical allodynia test in the diabetic neuropathy groups were significantly lower than those of the healthy groups (*P* < 0.05). On the other hand, in the ending weeks of the protocol implementation, the average changes of the mechanical allodynia test in the diabetic neuropathy training and training groups had a significant increase compared to those of the diabetic neuropathy group (*P* < 0.05) (see Figure 2).

**Figure 3:**
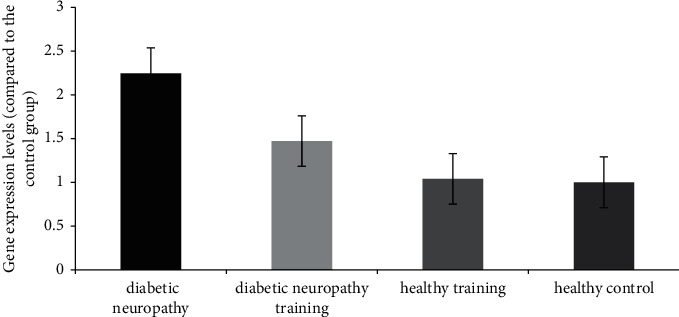
NLRP3 gene expression in the posterior region of the spinal cord of different groups.

Given the mean of the groups, it was found that induction of diabetes significantly increased the expression of the NLRP3 gene in the diabetic neuropathy rats compared to the healthy group (*P* < 0.05). Also, the expression of the NLRP3 gene in the diabetic neuropathy training group was significantly lower than that of the diabetic neuropathy group (*P* < 0.05) (see Figure 3).

**Figure 4:**
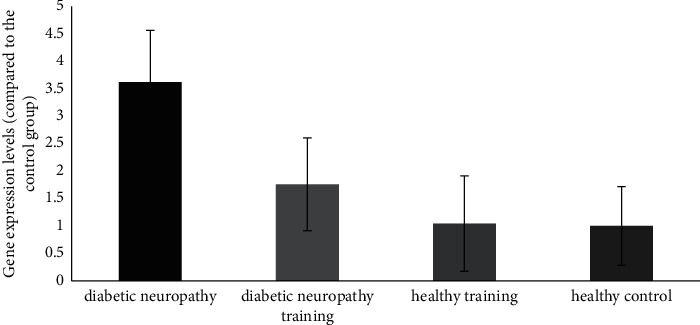
P38 MAPK gene expression in the posterior region of the spinal cord of different groups.

A significant increase in the P38 MAPK gene expression was observed in the posterior section of the spinal cord of the diabetic neuropathy group compared to the healthy control group (*P* ≤ 0.05). There was also a significant decrease in the diabetic neuropathy training group compared to the diabetic neuropathy group (*P* < 0.05) (see Figure 4).

**Figure 5:**
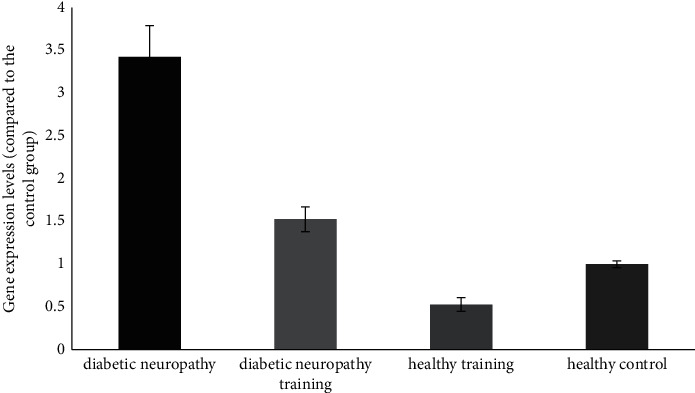
TNF-*α* gene expression in the posterior region of the spinal cord of different groups.

There was a significant increase in the TNF-*α* gene expression in the posterior section of the diabetic neuropathy group compared to the healthy control group (*P* < 0.05). There was also a significant decrease in the diabetic neuropathy training group compared to the diabetic neuropathy group (*P* < 0.05) (see Figure 5).

**Figure 6:**
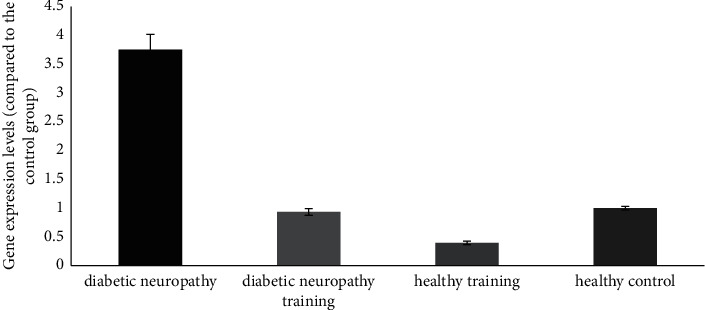
IL-1*β* gene expression in the posterior region of the spinal cord of different groups.

There was a significant increase in the IL-1*β* gene expression in the posterior section of the diabetic neuropathy group compared to the healthy control group (*P* < 0.05). There was also a significant decrease in the diabetic neuropathy training group compared to the diabetic neuropathy group (*P* < 0.05) (see Figure 6).

## 5. Discussion

In the present study, the effect of endurance training on the expression of NLRP3, P38 MAPK, TNF-*α*, and IL-1*β* genes in the spinal cord of rats with diabetic neuropathic pain was investigated. It was found that as a result of endurance training, the expression of these genes was reduced in the diabetic neuropathy training group compared to the diabetic neuropathy group and that this decrease was significant. In line with our findings, in a study to examine the effect of chronic and acute aerobic training with different intensities on inflammatory and signaling pathways of inflammasomes, Khakroo Abkenar et al. investigated the effect of various acute and chronic aerobic activities on the inflammatory pathways of NLRP3 inflammasomes, TLR4, and the levels of inflammatory cytokines such as IL-1*β* in young men. The results showed that chronic aerobic activity with moderate intensity leads to a downregulation pathway in the inflammasome pathway and inflammation, but acute aerobic activity with different intensities has no effect on inflammatory pathways [29]. Zhu et al., in their research, confirmed the upregulation in TNF-*α* inflammatory factors in the DRG and spinal cord in rats with diabetic neuropathic pain [30].

Also, consistent with the findings of the current research, Javid et al. examined the inhibitory effect of exercise on the activation of the NLRP3 inflammasome in obese rats. Obese rats performed eight weeks of training on the treadmill. Exercise and diet reduced overweight, fat accumulation, and insulin resistance in obese rats. In addition, it reduced the levels of gene and protein of inflammatory markers such as NLRP3 and caspase-1 in the adipose tissue. Also, in the isolated macrophages derived from bone marrow, it inhibited the activation of the NLRP3 inflammation pathway in the training group [31]. In another research, Sun et al. conducted a study on JMT, as a combined traditional Chinese medication, which is being used for many to treat diabetic neuropathic pain and examined the effect of 12 weeks of JMT consumption on NLRP3 in the posterior region of the spinal cord of diabetic rats. Behavioral changes were also examined by a mechanical pain threshold test. The results showed that JMT reduced the levels of mRNA and NLRP3 protein; also, the expression of IL-1*β* and caspase-1 in the spinal cord of diabetic neuropathy decreased. It was concluded that JMT can be used as an alternative to DNP therapy with a positive effect [32]. Boroujeni et al. examined the effect of nano-eugenol and aerobic exercise against streptozotocin toxicity and inflammatory mediators such as P38 MAPK in the dorsal root ganglia of diabetic rats. The results showed a significant decrease in the expression of the P38 MAPK gene in the diabetic rats that exercised and were treated with nano-eugenol compared to the normal model group. Accordingly, eugenol supplementation along with aerobic exercise is likely to control neuronal damage caused by diabetes [33]. It is likely that the mechanisms to explain the effects of endurance training on inflammatory factors can be attributed to the regulation of mTROS production with mitochondrial quality control (mitochondrial proliferation and mitochondrial activation), improvement of mitochondrial function, and increase in cleansing of damaged mitochondria, and hence, it can inhibit the NLRP3 inflammasome pathway and prevent extreme inflammatory reactions [34]. The nerve tissue has a frail antioxidant system, which due to high oxygen consumption is exposed to oxidative injuries; on the other hand, oxidative stress is the common pathway of cell damage as a result of hyperglycemia [35]. Endurance activity due to the inhibition of oxidative enzymes as well as due to antioxidant effects leads to improved intracellular defenses against ROS and thus reduces neural oxidation stress [36].

On the other hand, regular exercise by introducing cytosolic calcium and binding to the calmodulin receptor runs AKT/MTORC1 [37] and by consuming intramuscular glucose, releases insulin, increases IGF-1 function, and inhibits cellular inflammatory pathways [38] In addition, aerobic training can decrease the levels of inflammatory markers, prevent progressive degradation of sensory neurons, and reduce the sensitivity of nociceptors to pain factors by increasing the production of thermal shock proteins, reducing protein kinase C phosphorylation, increasing anti-inflammatory cytokines, reducing spinal cord microglial activity, and reducing free radical production [39–41]. In order to reduce the progression of neuronal damage in diabetes, it appears necessary to explore therapeutic options employing antioxidants and anti-inflammatory agents. Although pharmacological therapy methods have been proposed to date (e.g., nonsteroidal analgesics and opioids), none of them have been proven to be completely effective in treating or preventing diabetic neuropathic pain, and some of them may still have some side effects [42]. Hence, implementing methods that have no side effects or have minimal side effects, such as moderate endurance training, as well as nonpharmacologic and ancillary therapies, is of great significance.

## 6. Conclusion

Various neurodegenerative diseases, including chronic diabetic neuropathic pain, are associated with inflammation of the nervous system. The use of antioxidants and anti-inflammatory agents may be necessary to slow down the progression of diabetic neuronal damage. This study examined the effect of endurance training on the expression of NLRP3, P38 MAPK, TNF-*α*, and IL-1*β* genes in the spinal cord of rats with diabetic neuropathic pain. As a result of endurance training, there was a significant decrease in the expression of these genes in the diabetic neuropathy training group compared to the diabetic neuropathy group. Acute aerobic activity of varying intensities did not affect inflammatory pathways, but chronic aerobic activity of moderate intensity did. Furthermore, aerobic training can decrease markers of inflammation, slow the degradation of sensory neurons, and reduce the sensitivity of the nociceptors to certain types of pain factors through the production of thermal shock proteins, the reduction of protein kinase C phosphorylation, the production of anti-inflammatory cytokines, the reduction of spinal cord microglial activity, and the reduction of free radical production. Antioxidants and anti-inflammatory agents appear to be necessary for reducing the progression of neuronal damage in diabetes. There have been a number of attempts at pharmacological therapy (e.g., nonsteroidal analgesics, opioids), but none have been proven completely effective for treating or preventing diabetic neuropathic pain. Some of them may also have some side effects. A great deal of importance is thus attached to the implementation of methods that are side-effect-free or minimally side effect-free, such as moderate endurance training and other nonpharmacological therapies.

Combined findings from preclinical and clinical studies indicate that more research is needed. Randomized controlled studies are needed to convert preclinical knowledge to the clinical domain. Exercises that reduce neuropathic pain should be studied in depth for their method, intensity, frequency, and duration. It is important to integrate patient-reported and clinical results, as well as probable causes, into future research. Therefore, the review of research will likely lead to a consensus, and practitioners will be able to make more specific exercise recommendations to neuropathic pain patients.

## Figures and Tables

**Table 1:** Sequence of primers used in the present study.

Gens	Primer sequence
NLRP3	For: 5′- GGAGTGGATAGGTTTGCTGG -3′
	Rev: 5′- GGTGTAGGGTCTGTTGAGGT -3′

P38 MAPK	For: 5′- TTCATTCACAGCGAGGTTGC -3′
	Rev: 5′- GCTTACCGATGACCACGATC -3′

TNF-*α*	For: 5′- GAGATGTGGAAATGGCAGAGGA -3′
	Rev: 5′- GAGAAGATGATGTGAGTGTGAGG -3′

IL-1*β*	For: 5′-TGTGACTGGTGGGATGATGA -3′
	Rev: 5′-GTTCTGTCTATTGAGGTGGAGA -3′

GAPDH	For: 5′-GACATGCCGCCTGGAGAAAC-3′
	Rev: 5′-AGCCCAGGATGCCCTTTAGT-3′

**Table 2:** Mean and standard deviation of body weight and blood glucose levels in rats of different groups.

Variable		Groups			
		Diabetic neuropathy (*n* = 8)	Diabetic neuropathy + training (*n* = 8)	Healthy training (*n* = 8)	Healthy control (*n* = 8)
Weight (gr)	Induction of diabetes	205.13 ± 4.1	210.9 ± 6.6	199.11 ± 5.5	206.11 ± 8.4
	Week 2	198.10 ± 0.2	203.8 ± 3.7	203.10 ± 5.1	214.11 ± 6.1
	Week 4	185.10 ± 5.4	187.6 ± 4.9	216.9 ± 9.1	229.9 ± 3.1
	Week 6	160.8 ± 1.1	186.6 ± 0.4	227.8 ± 8.6	242.8 ± 8.1

Blood glucose (ml)	Induction of diabetes	421.113 ± 9.1	495.71 ± 1.6	114.31 ± 9.6	106.13 ± 8.8
	Week 2	465.81 ± 4.1	522.57 ± 4.9	144.16 ± 0	108.13 ± 1.8
	Week 4	515.61 ± 9.3	522.34 ± 4.8	108.14 ± 1.	102.14 ± 1.1
	Week 6	563.41 ± 4.0	434.55 ± 5.1	108.13 ± 3.1	101.13 ± 6.3

All values are presented as mean ± standard deviation.

## Data Availability

The data used to support the findings of this study are available and can be provided over email queries directly to the author at the corresponding address (taheriasma78@yahoo.com).
